# Obstetric Characteristics and Outcomes of Gestational Carrier Pregnancies: A Systematic Review and Meta-Analysis

**DOI:** 10.1001/jamanetworkopen.2024.22634

**Published:** 2024-07-23

**Authors:** Shinya Matsuzaki, Aaron D. Masjedi, Satoko Matsuzaki, Zachary S. Anderson, Katherine V. Erickson, Rachel S. Mandelbaum, Joseph G. Ouzounian, Richard J. Paulson, Koji Matsuo

**Affiliations:** 1 Department of Gynecology, Osaka International Cancer Institute, Osaka, Japan; 2 Department of Obstetrics and Gynecology, Osaka University Graduate School of Medicine, Suita, Japan; 3 Division of Gynecologic Oncology, Department of Obstetrics and Gynecology, University of Southern California, Los Angeles; 4 Department of Obstetrics and Gynecology, Osaka General Medical Center, Osaka, Japan; 5 Keck School of Medicine, University of Southern California, Los Angeles; 6 Department of Obstetrics and Gynecology, Division of Reproductive Endocrinology and Infertility, University of Southern California, Los Angeles; 7 Department of Obstetrics and Gynecology, Division of Maternal-Fetal Medicine, University of Southern California, Los Angeles; 8 Norris Comprehensive Cancer Center, University of Southern California, Los Angeles

## Abstract

**Importance:**

Advancements in assisted reproductive technology (ART) have led to an increase in gestational carrier (GC) pregnancies. However, the perinatal outcomes of GC pregnancies remain understudied, necessitating a deeper understanding of their associated risks.

**Objective:**

To assess maternal characteristics and obstetric outcomes associated with GC pregnancies.

**Data Sources:**

A comprehensive systematic search of publications published before October 31, 2023, using PubMed, Web of Science, Scopus, and Cochrane Library databases was conducted.

**Study Selection:**

Two authors selected studies examining obstetric characteristics and outcomes in GC pregnancies with 24 or more weeks’ gestation. Studies with insufficient outcome information, unavailable data on gestational surrogacies, and non-English language studies were excluded.

**Data Extraction and Synthesis:**

Adhering to Preferred Reporting Items for Systematic Reviews and Meta-Analysis guidelines, 2 investigators extracted and synthesized both quantitative and qualitative data. Both fixed-effect and random-effect analysis were used to pool data.

**Main Outcomes and Measures:**

The primary outcomes were obstetric characteristics and outcomes, including hypertensive disorders, preterm birth, and low birth weight. Secondary outcomes included severe maternal morbidity and mortality associated with GC pregnancies.

**Results:**

Six studies from 2011 to 2023 involving 28 300 GC pregnancies and 1 270 662 non-GC pregnancies were included. GCs accounted for 2.5% of in vitro fertilization cycles (59 502 of 2 374 154 cycles) and 3.8% of ART pregnancies (26 759 of 701 047 ART pregnancies). GC pregnancies were more likely to be conceived by frozen embryo transfer compared with non-GC ART pregnancies (odds ratio [OR], 2.84; 95% CI, 1.56-5.15), and rates of single embryo transfer were similar between the 2 groups (OR, 1.18; 95% CI, 0.94-1.48). GCs were rarely nulliparous (6 of 361 patients [1.7%]) and were more likely to have multifetal pregnancies compared with non-GC ART patients (OR, 1.18; 95% CI, 1.02-1.35). Comparator studies revealed lower odds of cesarean delivery (adjusted OR [aOR], 0.42; 95% CI, 0.27-0.65) and comparable rates of hypertensive disorders (aOR, 0.86; 95% CI, 0.45-1.64), preterm birth (aOR, 0.82; 95% CI, 0.68-1.00), and low birth weight (aOR, 0.79; 95% CI, 0.50-1.26) in GC pregnancies vs non-GC ART pregnancies. Comparatively, GC pregnancies had higher odds of hypertensive disorders (aOR, 1.44; 95% CI, 1.13-1.84) vs general (non-GC ART and non-ART) pregnancies with comparable cesarean delivery risk (aOR, 1.06; 95% CI, 0.90-1.25). Preterm birth and low birth weight data lacked a comparative group using multivariate analysis. Severe maternal morbidity and maternal mortality were rare among GCs.

**Conclusions and Relevance:**

In this systematic review and meta-analysis, although GC pregnancies had slightly improved outcomes compared with non-GC ART pregnancies, they posed higher risks than general pregnancies. Contributing factors may include ART procedures and increased rates of multiple gestations which influence adverse perinatal outcomes in GC pregnancies.

## Key Points

**Question:**

What are the obstetric characteristics and outcomes of gestational carrier pregnancies?

**Findings:**

This systematic review and meta-analysis evaluated 6 comparator studies involving 28 300 gestational carrier pregnancies and 1 270 662 non–gestational carrier pregnancies which showed that gestational carrier pregnancies had higher odds of hypertensive disorders than general pregnancies (ie, non–gestational carrier pregnancies with or without use of assisted reproductive technology) and comparable rates of preterm birth and low birth weight compared with non–gestational carrier pregnancies that used assisted reproductive technology. Severe maternal morbidity and maternal mortality were rare among gestational carriers.

**Meaning:**

These findings suggest that the risk profile of gestational carrier pregnancies shares similarities to other assisted reproductive technology–conceived pregnancies.

## Teaser

This systematic review and meta-analysis compares obstetric characteristics and outcomes of gestational carrier pregnancies with general pregnancies with and without the use of assisted reproductive technology.

## Introduction

Surrogacy involves a person carrying a fetus in utero and delivering a newborn for another person or people, termed the intended parents.^1^ It can be classified as traditional or gestational surrogacy, which may also be referred to as full or host surrogacy.^1^ Traditional surrogacy, now rare, uses the gametes (oocyte) of the surrogate, unlike gestational surrogacy,^2^ in which an embryo from donated gametes or from the intended parents is implanted into a gestational carrier’s (GC) uterus. The increasing number of GCs is attributed to increased awareness of GC pregnancies, broader access to assisted reproductive technology (ART) services, and evolving surrogacy regulations.^3^

In the US, gestational surrogacy is recommended if the intended parents face biological, medical, ethical, or psychosocial obstacles to pregnancy.^4^ Some common indications for use of a GC include a biological inability to carry or gestate a pregnancy, a congenital absence of the uterus, or an acquired absence of the uterus secondary to cancer treatment, disease management, or obstetrical hemorrhage.^5^ Additionally, in cases where an unknown endometrial factor hinders successful ART despite high-quality embryos, a GC may be considered in certain countries. Finally, many individuals with medical contraindications to pregnancy (eg, cervical insufficiency, autoimmune disease, or cardiac disease) may use a GC.

GCs may experience the potential risks associated with pregnancy and delivery, such as hypertensive disorders of pregnancy (HDP), gestational diabetes (GD), or cesarean delivery (CD) due to the high rate of multiple gestations, which is an iatrogenic complication of ART. ART for GCs may be associated with increased rate of placenta previa, placenta accreta spectrum, and placental abruption.^6,7^ The objective of this study was to assess maternal characteristics and obstetric outcomes associated with GC pregnancies. Thus, we conducted a comprehensive systematic review and meta-analysis to assess these outcomes and gather information which is crucial for patient counseling, obtaining informed consent, and for identifying suitable candidates to be a GC.^4^ We hypothesized that GC pregnancies would have worse pregnancy outcomes, including higher rates of HDP, preterm birth (PTB), and low birth weight (LBW) due to the increased rate of multiple pregnancies compared with general pregnancies.

## Methods

### Literature Review

This systematic review and meta-analysis was registered with the International Prospective Register of Systematic Reviews.^8^ Because the current systematic review used publicly available and deidentified data, the Osaka International Cancer Institution institutional review board exempted the present study and the requirements of informed patient consent. This study followed the Preferred Reporting Items for Systematic Reviews and Meta-Analyses (PRISMA) reporting guideline.^9^

### Eligibility Criteria, Information Sources, and Search Strategy

Following prior methodologies, a systematic search of publications published before October 31, 2023, covered PubMed, Scopus, Web of Science, and the Cochrane Central Register of Controlled Trials using specific terms (eAppendix 1 in Supplement 1).^10,11,12^ Titles, abstracts, and full texts were screened by 2 investigators (Shinya Matsuzaki and Satoko Matsuzaki). From this set, studies exploring the associations of GC pregnancy with relevant outcomes were extracted using keywords such as *surrogate mothers* (medical subject heading terms) or related keywords of surrogate mothers and pregnancy outcome (medical subject heading terms) or related keywords of pregnancy outcomes.

### Study Selection

Study selection adhered to the patient population, intervention, comparator, outcome, and study type (PICOS) design (eAppendix 2 in Supplement 1).^9^ The study inclusion criteria were (1) pregnancy outcomes in gestational surrogacy, (2) studies comparing obstetric outcomes between gestational surrogacy and nonsurrogacy, and (3) pregnancies with 24 or more weeks’ gestation. The exclusion criteria comprised (1) insufficient outcome information; (2) unavailable data on the number of gestational surrogacies; (3) non-English language studies; and (4) conference abstracts, editorials, case reports, case series, narrative reviews, systematic reviews, and meta-analyses.

### Data Extraction

Data were extracted by 2 investigators (Shinya Matsuzaki and Satoko Matsuzaki), who recorded the study year, location, first author’s name, number of cases, and the relevant outcomes. Pregnant individuals were classified into 3 groups: (1) GC pregnancies, (2) non-GC ART pregnancies, and (3) non-GC non-ART pregnancies. In this study, non-GC pregnancies were defined as non-GC ART and/or non-GC non-ART pregnancies, and general pregnancies included both non-GC ART and non-GC non-ART pregnancies.

### Outcome Measure Analysis and Assessment of Risk of Bias

The 2 primary outcomes were maternal characteristics and obstetric outcomes in GC pregnancies. Areas of interest included HDP, GD, fetal growth restriction, PTB, LBW, intrauterine fetal death, placenta previa, and placental abruption. In the sensitivity analysis, the characteristics of ART treatment of GC pregnancies were explored.

Secondary outcomes included severe maternal morbidity (SMM) and delivery outcomes, such as the rate of CD and postpartum hemorrhage. SMM was based on definitions by the Centers for Disease Control and Prevention (including eclampsia, blood transfusion, and hysterectomy),^13^ and modified to include maternal death; intensive care unit admission; and hemolysis, elevated liver enzymes, and low platelets (HELLP) syndrome. A composite of SMMs determined in each study was used for analysis. Risk of bias assessment employed the Risk of Bias in Nonrandomized Studies of Interventions Tool (ROBINS-I).^14,15,16^

### Meta-Analysis Plan

Maternal outcome risks were estimated from the eligible studies in experimental and control groups using 95% CIs of reported values to derive odds ratios (ORs). Studies that did not provide raw data were excluded; the majority of studies presented ORs. Study heterogeneity was assessed using *I^2^* percentages and a fixed- or random-effect analysis was performed as shown in eAppendix 3 in Supplement 1. Data from continuous and bivariate outcomes were entered for consistency, favoring active interventions due to negative effect sizes or relative risks less than 1. Any adjusted results were based on adjustments that were defined by the original studies to account for confounding variables.

### Statistical Analysis

Baseline demographic differences between groups were assessed using the χ^2^ or Fisher exact test. Meta-analysis and visualizations were performed using RevMan software version 5.4.1 (Cochrane Collaboration). Statistical analyses were also conducted with SPSS version 28.0 (IBM). A 2-sided *P* < .05 was considered statistically significant.

## Results

### Study Selection

**Figure 1.**
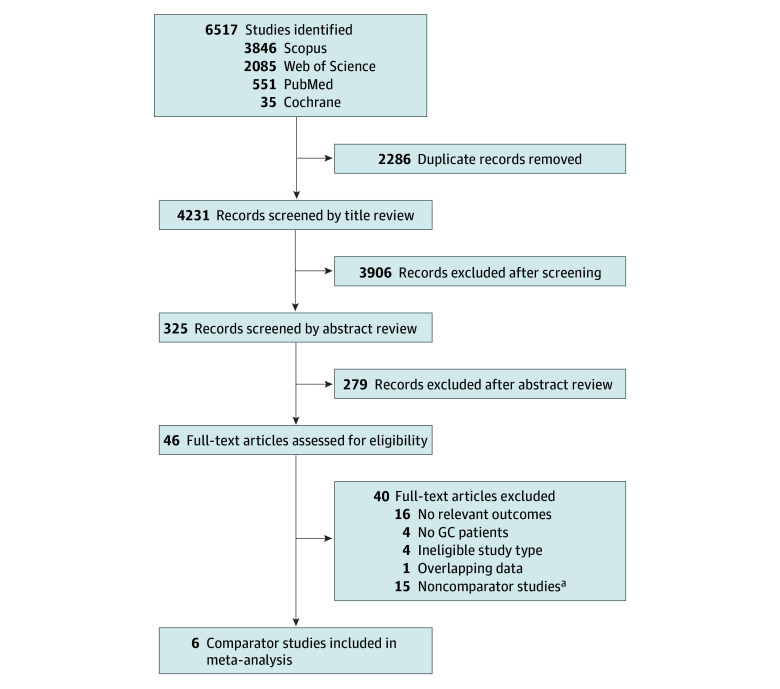
Study Selection Scheme of the Systematic Literature Search GC indicates gestational carrier. ^a^Descriptive statistics of noncomparator studies are described in eTable 2, eTable 3, and eTable 4 in Supplement 1.

Of 4231 studies reviewed, 22 studies reported the obstetric outcomes of GC pregnancies (Figure 1 and eTable 1 in Supplement 1).^3,17,18,19,20,21,22,23,24,25,26,27,28,29,30,31,32,33,34,35,36,37^ Two studies with overlapping data were identified,^22,23^ and the older study was excluded from the descriptive analysis.^23^ Fifteen noncomparator studies were excluded from the main comparator analysis (eTables 2-4 in Supplement 1).^18,19,20,21,24,26,27,29,31,32,33,34,35,36,37^ As a result, 6 studies involving 28 300 GC pregnancies and 1 270 662 non-GC pregnancies underwent further descriptive analysis (Figure 1).^3,17,22,25,28,30^

### Study Characteristics

All 6 studies^3,17,22,25,28,30^ were retrospective studies published between 2011 and 2023 (no randomized clinical studies). The majority of the studies (5 studies [83.3%]) originated from the US^3,17,22,25,30^ and 1 study (16.7%) was from the UK.^28^ Among the 6 studies, 1 compared obstetric outcomes between GC pregnancies and general pregnancies,^22^ and all compared the outcomes between GC pregnancies and non-GC ART pregnancies (Table 1).^3,17,22,25,28,30^

**Table 1.** Characteristics of Patients With and Without GCs at ART Treatment

Study characteristic	Participants by study, No./total No. (%)					
	Shandley et al,^17^ 2023	Swanson et al,^22^ 2021	Segal et al,^25^ 2018	Sunkara et al^28^ 2017	Perkins et al,^3^ 2016	Gibbons et al,^30^ 2011
Location	US	US	US	UK	US	US
Data source	SART and CORS	Utah	SART	HFEA	NASS	SART and CORS
Duration	2014-2020	2009-2018	2014	1996-2011	2009-2013	2004-2006
Type	Retrospective	Retrospective	Retrospective	Retrospective	Retrospective	Retrospective
Comparison	GC vs ART	GC vs ART vs general	GC vs IP	GC vs ART	GC vs ART	GC vs ART
Total cycles, No.						
GC	40 177	NA	1927	1536	14 682	1180
ART	968 028	NA	NA	624 319	633 775	70 213
General	NA	NA	NA	NA	NA	NA
IP	NA	NA	18 317	NA	NA	NA
Age >35 y^a^						
GC	12 642/40 177 (31.5)	NA	407/1927 (21.1)	713/1536 (46.4)	1103/14 682 (7.5)	NA
ART	562 358/968 028 (58.1)	NA	NA	334 462/624 319 (53.6)	372 712/633 775 (58.8)	NA
General	NA	NA	NA	NA	NA	NA
IP	NA	NA	15 787/18 317 (86.2)	NA	NA	NA
Nulliparous^a^						
GC	NA	NA	NA	NA	NA	NA
ART	620 838/968 028 (64.1)	NA	NA	559 054/624 319 (89.5)	418 440/633 775 (66.0)	NA
General	NA	NA	NA	NA	NA	NA
IP	NA	NA	NA	NA	NA	NA
ART type						
Fresh ET						
GC	3912/40 177 (9.7)	NA	754/1927 (39.1)	NA	7539/14 682 (51.4)	NA
ART	328 761/968 028 (34.0)	NA	NA	505 186/624 319 (80.9)	444 084/633 775 (70.1)	NA
General	NA	NA	NA	NA	NA	NA
IP	NA	NA	10 657/18 317(58.2)	NA	NA	NA
Frozen ET						
GC	36 211/40 177 (90.1)	NA	1176/1927 (61.0)	NA	7143/14 682 (48.7)	NA
ART	637 691/968 028 (65.9)	NA	NA	119 133/624 319 (19.1)	189 691/633 775 (29.9)	NA
General	NA	NA	NA	NA	NA	NA
IP	NA	NA	7670/18 317 (41.9)	NA	NA	NA
ET, mean (SD)						
GC	NA	NA	1.5 (0.7)	1.7 (0.7)	NA	NA
ART	NA	NA	NA	1.8 (0.8)	NA	NA
General	NA	NA	NA	NA	NA	NA
IP	NA	NA	1.4 (0.7)	NA	NA	NA
Single ET						
GC	29 096/40 177 (72.4)	NA	578 (74.4)^b^	NA	2281/14 682 (15.5)^b^	NA
ART	619 947/968 028 (64.0)	NA	NA	NA	86 537/633 775 (13.7)^b^	NA
General	NA	NA	NA	NA	NA	NA
IP	NA	NA	5726/NA (78.5)^b^	NA	NA	NA

Abbreviations: ART, assisted reproductive technology; CORS, Clinic Outcome Reporting System; ET, embryo transfer; GC, gestational carrier; HFEA, Human Fertilization and Embryology Authority; IP, ART pregnancies of intended parents; NA, not applicable; NASS, National ART Surveillance System; SART, The Society for Assisted Reproductive Technology; Utah, Utah Department of Health Office of Vital Records and Statistics.

^a^ Status at ART treatment.

^b^ Elective single embryo transfer.

In the 6 eligible studies, age at ART treatment and ART type (frozen or fresh embryo transfer) were reported in 4 studies^3,17,25,28^ and rate of single embryo transfer was reported in 3 studies^3,17,25^; prior live birth was not reported in any study (Table 1). The method of endometrial preparation was unavailable in all studies. Maternal age and nulliparity were specified in 1 study,^22^ and multiple pregnancy rates were specified in 5 studies^3,17,22,25,28^ (Table 2).

**Table 2.** Maternal Outcomes of GC Pregnancies

Study characteristic	Outcome by study, OR (95% CI)						
	Shandley et al, ^17^ 2023	Swanson et al,^22^ 2021		Segal et al,^25^ 2018	Sunkara et al,^28^ 2017	Perkins et al,^3^ 2016	Gibbons et al^30^ 2011
Location	US	US	US	US	UK	US	US
Comparison	GC vs ART^a^	GC vs general	GC vs ART^a^	GC vs IP^a^	GC vs ART^a^	GC vs ART^a^	GC vs ART^a^
Participants, No.	441 905^b^	509 376^b^	509 376^b^	8384^b^	103 160^b^^,^^c^	174 357^b^	71 393^d^
GC pregnancies, No.	21 649^b^	361^b^	361^b^	1009^b^/716^b^^,^^c^	244^b^^,^^c^	3857^b^	1180^d^
Control participants, No.	420 256^b^	509 015^b^	563^b^	7375^b^/5634^b^^,^^c^	102 916^b^^,^^c^	170 500^b^	60 037^d^
Age, median (IQR), y	NA	NA	31 (28-34) in GC vs 38 (33-43) in ART	NA	NA	NA	NA
Nulliparous, No./Total No. (%)							
GC	NA	6/361 (1.7)	6/361 (1.7)	NA	NA	NA	NA
ART	NA	NA	350/563(62.2)	NA	NA	NA	NA
General	NA	166 441/509 015 (32.7)	NA	NA	NA	NA	NA
Multiple pregnancies, No./Total No. (%)							
GC	3197/21 649 (14.8)	77/361 (21.3)	77/361 (21.3)	293/1009 (29.0)	71/325 (21.8)	1453/3857 (37.7)	NA
ART	52 957/420 256 (12.6)	NA	146/563(25.9)	NA	32 321/138 327 (23.4)	50 554/170 550 (29.7)	NA
General	NA	8881/509 015 (1.7)	NA	NA	NA	NA	NA
IP	NA	NA	NA	1740/7375 (23.6)	NA	NA	NA
Maternal complication							
Unadjusted HDP	NA	1.84 (1.31-2.59)	0.42 (0.28-0.63)	NA	NA	NA	NA
Adjusted HDP	NA	1.44 (1.13-1.84)^e^	0.86 (0.45-1.64)^f^	NA	NA	NA	NA
Unadjusted PTB	0.97 (0.94-1.01)	NA	NA	0.62 (0.51-0.76)^c^	0.89 (0.56-1.41)^c^	1.27 (1.18-1.36)	NA
Adjusted PTB	NA	NA	NA	0.78 (0.61-0.99)^c^^,^^g^	0.92 (0.66-1.30)^c^^,^^h^	NA	NA
Unadjusted LBW	NA	NA	NA	0.49 (0.36-0.67)^c^	0.87 (0.55-1.38)^c^	1.09 (1.01-1.17)	0.84 (0.68-1.04)^i^
Adjusted LBW	NA	NA	NA	0.62 (0.44-0.89)^c^^,^^g^	1.01 (0.71-1.42)^c^^,^^h^	NA	NA
Unadjusted CD	NA	1.21 (0.96-1.54)	0.22 (0.17-0.30)	NA	NA	NA	NA
Adjusted CD	NA	1.06 (0.90-1.25)^e^	0.42 (0.27-0.65)^j^	NA	NA	NA	NA
Unadjusted mortality	NA	2.74 (0.17-44.06)^f^	NA	NA	NA	NA	NA
Unadjusted SMM	NA	1.61 (0.72-3.60)	0.29 (0.12-0.70)	NA	NA	NA	NA
Adjusted SMM	NA	1.03 (0.51-2.07)^e^	0.17 (0.04-0.81)^j^	NA	NA	NA	NA

Abbreviations: ART, assisted reproductive technology; CD, cesarean delivery; GC, gestational carrier; HDP, hypertensive disorders of pregnancy; IP, ART pregnancies of intended parents; LBW, low birth weight; NA, not applicable; OR, odds ratio; PTB, preterm birth; SMM, severe maternal morbidity.

^a^ Non-GC ART pregnancies.

^b^ Number of pregnancies with live births.

^c^ Restricted to singleton pregnancies.

^d^ Number of ART cycles.

^e^ Adjusted for age, nulliparity, and tobacco.

^f^ Estimated with RevMan version 5.4.1.

^g^ Model adjusted for age, use of assisted hatching, intracytoplasmic sperm injection, preimplantation genetic diagnosis, fresh or frozen donor oocyte, and number of embryos transferred, reduction in fetal heart.

^h^ Adjusted for female age category, period of treatment, number of previous in vitro fertilization cycles, previous live birth occurrence, cause of infertility, mean number of embryos transferred, and initial singleton or multiple pregnancies that lead to singleton live births.

^i^ Comparison with patients conceived by frozen embryo transfer.

^j^ Adjusted for age, nulliparity, chronic hypertension, and substance use.

### Risk of Bias of Included Studies

Among the 6 identified comparator studies (GC pregnancies vs non-GC pregnancies), risk of bias assessments were conducted. There was moderate bias (moderate quality) in 4 studies^3,17,22,28^and severe bias (low quality) in 2 studies^25,30^ (eTable 5 in Supplement 1).

### Measured Outcomes

The following relevant outcomes were assessed in the 6 comparator studies: HDP (1 study),^22^ GD (0 studies), PTB (4 studies),^3,17,25,28^ LBW (4 studies),^3,25,28,30^ CD (1 study),^22^ maternal mortality (1 study),^22^ and SMM (1 study).^22^ Adjusted ORs (aORs) of obstetric outcomes in multivariate analyses were detailed for HDP (1 study),^22^ PTB (2 studies),^25,28^ LBW (2 studies),^25,28^ and CD (1 study).^22^

### ART Treatment for GC Pregnancies

**Figure 2.**
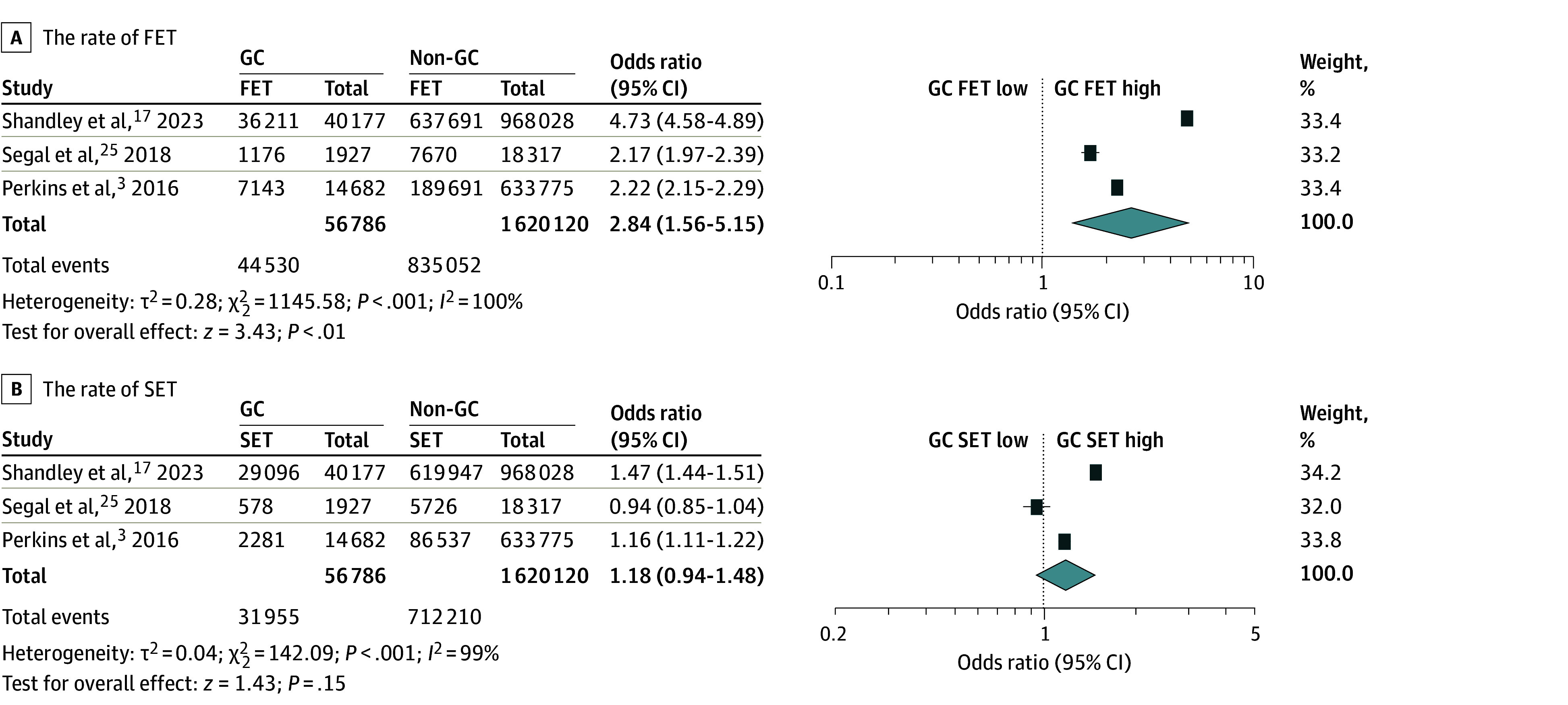
Meta-Analysis for the Assisted Reproductive Technology (ART) Treatment of Gestational Carrier (GC) Pregnancies The pooled odds ratio for unadjusted analysis for frozen embryo transfer (FET; A) and unadjusted analysis for single embryo transfer (SET; B) between GC pregnancies vs non-GC ART pregnancies. Heterogeneity among the studies in each analysis was defined as considerable heterogeneity in unadjusted random-effect analysis. Some values listed in the figure might be slightly different from the original values because of the calculation in RevMan version 5.4.1.

The cumulative rate of GC in vitro fertilization cycles was 2.5% (59 502 of 2 374 154 cycles) among ART cycles. GC pregnancies were more likely to be conceived by frozen embryo transfer than non-GC ART pregnancies (OR, 2.84; 95% CI, 1.56-5.15), whereas the use of single embryo transfer was similar between the 2 groups (OR, 1.18; 95% CI, 0.94-1.48) (Figure 2).

### Rate of GC Pregnancies

Among the 6 comparator studies, 1 clarified the number of GC pregnancies among general pregnancies.^22^ Four studies clarified the number of GC pregnancies among ART pregnancies,^3,17,25,28^ and 2 studies were excluded because the total number of ART pregnancies was not clarified (Table 1).^22,30^ Based on this data, GC pregnancies comprised approximately 3.8% of ART pregnancies (26 759 of 701 047 ART pregnancies) and 0.1% of all pregnancies (361 of 509 376 pregnancies).

### Patient Characteristics

Of the 6 comparator studies, 1 compared maternal age between GC pregnancies and non-GC ART pregnancies (Table 2).^22^ The median (IQR) maternal age was lower in GC pregnancies (31 [28-34] years) than in non-GC ART pregnancies (38 [33-43] years]) (*P* < .001). There was a lower prevalence of nulliparity among GC pregnancies (6 of 361 [1.7%]) than among general pregnancies (166 441 of 509 015 [32.7%]) (*P* < .001); there was also a lower prevalence of nulliparity among GC pregnancies than or non-GC ART pregnancies (350 of 563 [62.2%]) (*P* < .001). In the noncomparator studies,^18,19,20,21,24,26,27,29,31,32,33,34,35,36,37^ the cumulative mean (range) maternal age of GCs was 34.2 (32.7-38.8) years and the rate of nulliparity was 1.0% (12 of 1222 patients; range 0.1%-3.0%).

Five studies^3,17,22,25,28^ included information on multiple gestation (ranging from 3197 of 21 649 pregnancies [14.8%] to 1453 of 3857 pregnancies [37.7%]). One study^22^ reported a significantly higher rate of multiple gestation in GC pregnancies as compared with general pregnancies (OR, 15.27; 95% CI, 11.86-19.66). When compared with non-GC ART pregnancies, patients with GC pregnancies were more likely to have multiple gestation (OR, 1.18; 95% CI, 1.02-1.35).^3,17,22,25,28^ In the noncomparator studies,^18,19,20,21,24,26,27,29,31,32,33,34,35,36,37^ the cumulative rate of multiple gestation (excluding singleton-restricted studies) reached 21.6% (443 of 2055 patients; range 0.0%-34.7%).

### HDP

One comparator study^22^ compared HDP risks between GC pregnancies and general pregnancies and between GC pregnancies and non-GC ART pregnancies (Table 2). This study found more HDP in GC pregnancies than in general pregnancies (aOR, 1.44; 95% CI, 1.13-1.84) and similar HDP risk between GC pregnancies and non-GC ART pregnancies (aOR, 0.86, 95% CI, 0.45-1.64).^22^ Singleton deliveries–specific analysis was unavailable for HDP.

### PTB

**Figure 3.**
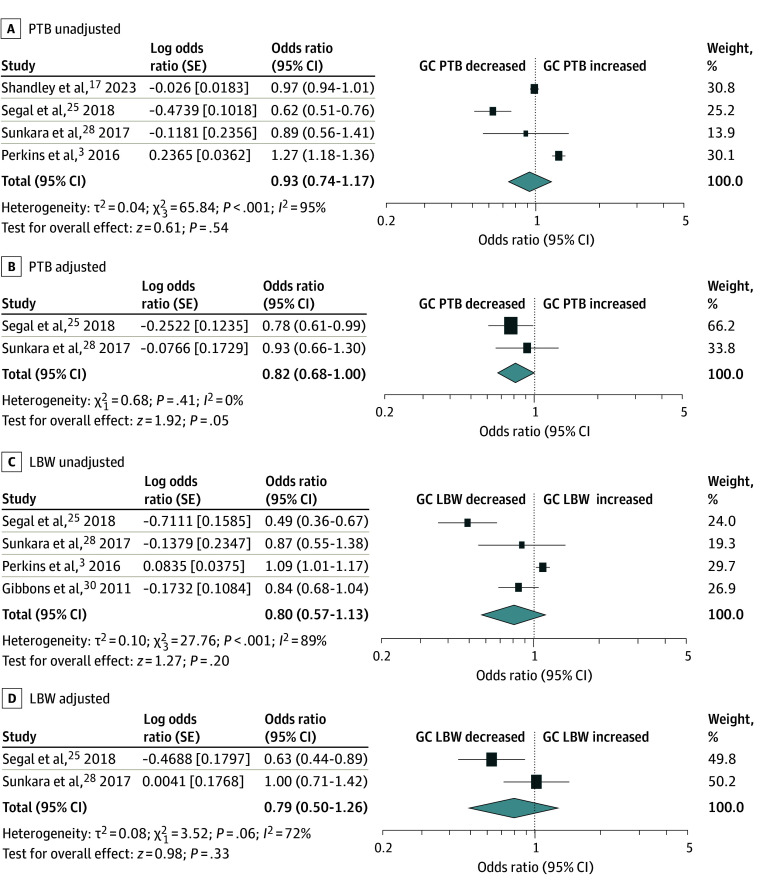
Meta-Analysis for the Association of Gestational Carrier (GC) Pregnancies With Preterm Birth (PTB) and Low Birth Weight (LBW) The pooled odds ratios are shown for unadjusted analysis for PTB (A), adjusted analysis for PTB (B), unadjusted analysis for LBW (C), and adjusted analysis for LBW (D) for GC pregnancies vs non-GC pregnancies. Heterogeneity among the studies in each analysis was defined as considerable heterogeneity in unadjusted random-effect analysis (A, C), no heterogeneity in adjusted fixed-effect analysis (B), and substantial heterogeneity in adjusted random effect analysis (D). Some values listed in the figure might be slightly different from the original values because of the calculation in RevMan version 5.4.1.

PTB was assessed in 4 of 6 comparator studies.^3,17,25,28^ In the pooled analysis, PTB risks were comparable between GC pregnancies and non-GC ART pregnancies, evident in both unadjusted random-effects (OR, 0.93; 95% CI, 0.74-1.17; *I^2^* = 95%; χ^2^_3_ = 65.84; *P* < .001) and adjusted fixed-effects analyses (available only for singleton pregnancies restricted data in 2 studies^25,28^) (aOR, 0.82; 95% CI, 0.68-1.00; *I^2^* = 0%; χ^2^_1_ = 0.68; *P* = .41) (Figure 3).

### LBW

Four comparator studies assessed LBW in GC pregnancies.^3,25,28,30^ The pooled analysis indicated comparable LBW risk between GC pregnancies and non-GC ART pregnancies in both unadjusted random-effects (OR, 0.80; 95% CI, 0.57-1.13; *I^2^* = 89%; χ^2^_3_ = 27.76; *P* < .001)^3,25,28,30^ and adjusted random-effects analyses (available only for singleton pregnancies restricted data in 2 studies^25,28^), but the adjusted analysis was not statistically significant (aOR, 0.79; 95% CI, 0.50-1.26; *I^2^* = 72%; χ^2^_1_ = 3.52; *P* = .06) (Figure 3).

### CD Risk

A nationwide study in the US investigated the association of GC pregnancies with CD risk.^22^ In this unadjusted analysis, GC pregnancies had similar CD risk compared with general pregnancies (OR, 1.21; 95% CI, 0.96-1.54) but a lower CD risk compared with non-GC ART pregnancies (OR, 0.22; 95% CI, 0.17-0.30) (Table 2). These findings were consistent in multivariate analysis, indicating comparable CD rates between GC pregnancies and general pregnancies (aOR, 1.06; 95% CI, 0.90-1.25) and lower CD risks in GC pregnancies compared with non-GC ART pregnancies (aOR, 0.42; 95% CI, 0.27-0.65). Singleton-specific analysis was unavailable for CD.

### SMM and Maternal Mortality

SMM was determined in 1 comparator study^22^ that assessed the composite evaluation of intensive care unit admission, eclampsia, HELLP, transfusion, and hysterectomy. The composite risk of SMM was similar between GC pregnancies and general pregnancies (aOR, 1.03; 95% CI, 0.51-2.07), whereas the composite risk was lower in GC pregnancies compared with non-GC ART pregnancies (aOR, 0.17; 95% CI, 0.04-0.81). Maternal mortality was assessed in 1 comparator study,^22^ which included 361 GC pregnancies with no maternal deaths, whereas 256 of 509 015 cases of maternal death (0.1%) were seen in general pregnancies (OR, 2.74; 95% CI, 0.17-44.06) (Table 2).

## Discussion

The results from this systematic review and meta-analysis demonstrated 4 principal findings. First, GC pregnancies represented 3.8% of ART pregnancies and 0.1% of all pregnancies. Second, although there was insufficient evidence on obstetric outcomes of GC pregnancies, especially regarding SMM, these pregnancies often involve multiparous patients and a high rate of multiple gestations. Third, obstetric outcomes of GC pregnancies, excluding CD and SMM, were similar to those seen in ART pregnancies but could result in worse outcomes compared with unassisted pregnancies. Fourth, GC pregnancies may have higher HDP risks than non-GC pregnancies. While some findings aligned with existing knowledge, the scarcity of comparator studies in prospective settings underscores the need for further investigation.

### Primary Outcomes: Maternal Characteristics and Obstetric Outcomes

Pregnancies in multiparous patients with a history of successful, uncomplicated term pregnancies are typically lower risk.^38^ However, studies consistently found associations of ART pregnancies with higher risk of adverse obstetric outcomes and multiple gestation compared with non-ART conceptions.^39,40,41^ Notably, the cause of infertility further elevates risk of adverse obstetric outcomes during ART pregnancies, particularly with multiple gestation, leading to increased chances of PTB, HDP, and LBW.^42,43,44^ Thus, ART and multiple gestation may be the main factors associated with increased risk for GC pregnancies, whereas multiparous patients without infertility and a history of uncomplicated pregnancies tend to have good prognosis.

A retrospective study^27^ showed higher odds of twin pregnancies, PTB, GD, placental previa, and CD in GC pregnancies (103 patients) vs the GC’s own prior pregnancies (294 patients). Another 2020 study in the US with a limited sample size found similar obstetric outcomes (PTB, GD, postpartum hemorrhage, fetal growth restriction, placental abruption, and abnormal placentation) when comparing a GC singleton pregnancy (78 patients) with their own prior singleton pregnancies (71 patients).^37^

A retrospective study^21^ that compared the obstetric outcomes between GCs with singleton gestation (284 pregnancies) and multiple gestation (77 pregnancies) showed that GC pregnancies with multiple gestation had increased odds of PTB compared with GC pregnancies with singleton gestation (aOR, 29.3; 95% CI, 11.0-78.0) and CD (aOR, 5.6; 95% CI, 3.1-10.2). The poorer prognosis of GC pregnancies may primarily stem from higher rates of multiple gestation. Therefore, efforts to reduce multiple gestation, such as elective single embryo transfer, are crucial to improving the obstetric outcomes of GC pregnancies.

In the present study, PTB and LBW risks were comparable between GC pregnancies and non-GC ART pregnancies, but no studies included non-GC non-ART controls. One study^22^ found lower CD risks but similar HDP risks in GC pregnancies than in non-GC ART pregnancies. The increased HDP following oocyte donation is theorized to be caused by an immunological maladaptation to the foreign antigens from the fetus.^28,45^ Gestational surrogacy involves carrying a pregnancy that is the result of either the intended parent’s or parents’ gametes, or donor gametes.^28^ Theoretically, immune reactions to foreign antigens in gestational surrogacy may be comparable with responses observed in people after receiving oocyte donations, which increases the risk of HDP compared with autologous ART.^28,45^

Another potential factor that could increase the risk of HDP in GC pregnancies is the widespread use of frozen embryo transfer. In this study, GCs were more likely to conceive with frozen embryo transfer than non-GCs. A meta-analysis of 3 randomized trials^46^ involving 1193 pregnancies after frozen embryo transfer and 1205 after fresh embryo transfer showed an increased risk of HDP after frozen embryo transfer compared with fresh embryo transfer. A population-based study in Norway^47^ reported a comparable HDP risk between fresh embryo transfer and natural conception (aOR, 1.02; 95% CI, 0.98-1.07), whereas frozen embryo transfer was associated with an increased HDP risk compared with natural conception (aOR, 1.74; 95% CI, 1.61-1.89). Although the type of endometrial preparation was unavailable in this study, these data are suggestive in that frozen embryo transfer use in GCs could potentially increase the risk of HDP.

Given limited information regarding patient characteristics in GCs, descriptive statistics of noncomparator studies are summarized in eTables 2 to 4 in Supplement 1.^18,19,20,21,24,26,27,29,31,32,33,34,35,36,37^ In the noncomparator studies, the cumulative mean maternal age of GCs, the cumulative rate of multifetal gestation, and the rate of nulliparity results may be consistent with comparator studies and enhance the robustness of the results of the meta-analysis; however, it could not be fully determined because the reported outcomes were different between the comparator and noncomparator studies.

### Secondary Outcomes: SMM and Delivery Outcomes

The American Society for Reproductive Medicine (ASRM) committee recommends selecting GCs who are aged 21 to 45 years, with at least 1 previous term delivery without complications, less than 5 previous deliveries, less than 3 previous CDs, and a stable family environment.^4^ In adherence to these recommendations, GC pregnancies will have the following characteristics: (1) fertile multiparous patients with previous uncomplicated deliveries, (2) younger maternal age, (3) normal psychological evaluation, and (4) normal medical evaluation. Although GCs have these characteristics, GC pregnancies are a result of ART, which has a known risk of multiple gestation pregnancy. Given the notable risks described throughout the data, gestational surrogates should undergo rigorous screening and testing to determine appropriate candidates.

SMM was examined in only 1 comparator study^22^ that was underpowered due to the limited number of included GC pregnancies (361 patients). Thus, the risk of SMM in GC pregnancies is still unknown. More extensive studies are needed to examine the association of GC pregnancies with SMM. Notably, 1 study^23^ evaluated outcomes in GC pregnancies in patients who did not match the ASRM safety guidelines. The outcomes showed a correlation with severe obstetric and neonatal complications. Although SMM was rare in these GC pregnancies that did not abide by ASRM guidelines, these pregnancies did have higher rates of CD, neonatal morbidity, and PTB. Thus, careful GC candidate selection may substantially improve obstetric outcomes.

### Limitations

This study had several limitations. First, there was inherent bias from the inclusion of retrospective studies as well as confounding variables. Second, no included studies comprehensively analyzed patient obstetric history to investigate the association of gestational surrogacy with obstetric outcomes. Consequently, a causal relationship between gestational surrogacy and maternal outcomes could not be established. Third, publication bias remains a substantial concern, potentially skewing findings toward positive associations of maternal outcomes with gestational surrogacy.

Fourth, there are limited comparative studies examining maternal outcomes in gestational surrogacy, requiring more comprehensive investigations. Moreover, eligible studies of the current systematic review were missing key outcome variables, thus limiting the ability to perform meaningful meta-analyses and limiting the conclusiveness and impact of the study. This weakness was due to the limited available obstetric outcomes of the data sources used in the eligible studies. Given the challenges associated with conducting a randomized clinical trial, a prospective study may be appropriate.

Fifth, data on previous pregnancies were absent, but a history of PTB, HDP, or CD is associated with an increased rate of subsequent pregnancy complications. Finally, psychological and physical evaluations for gestational surrogates could decrease obstetric complications, potentially introducing selection bias. Acknowledging this limitation is crucial when interpreting the results of the current study.

Sixth, although multiple gestations are associated with adverse outcomes, the outcomes controlling for gestation in the analysis were unavailable because of the lack of data in the eligible studies. Elective single embryo transfer is recommended to decrease multiple gestation in ART pregnancies, especially in GC pregnancies. In alignment with the nationwide push promoting single embryo transfer, it is likely that some of these outcomes have improved over time. Nevertheless, these data were not available for the current systematic review and further research considering these factors is warranted to improve the obstetric outcomes of GC pregnancies.

## Conclusions

In this systematic review and meta-analysis of characteristics and maternal outcomes of GC pregnancies, we found comparable obstetric outcomes between GC pregnancies and non-GC ART pregnancies, but a lack of comparisons with non-GC non-ART pregnancies persists. There is a need for further research to comprehensively understand obstetric outcomes in GC pregnancies and better understand the associated risk profile.

## Notes

Supplement 1. **eAppendix 1.** Searching Keywords **eAppendix 2.** PICOS Criteria for Inclusion of Systematic Review **eAppendix 3.** The Definition of Heterogeneity **eTable 1.** Metadata of Comparator and Non-Comparator Studies **eTable 2.** Maternal Characteristics of Gestational Carrier Pregnancies (Non-Comparator Studies) **eTable 3.** Obstetric Outcomes Among Gestational Carrier Pregnancies (Non-Comparator Studies) **eTable 4.** Obstetric Outcomes in Singleton Gestational Carrier Pregnancies (Non-Comparator Studies) **eTable 5.** Risk of Bias Assessment for the Comparator Study
eReferences.

Supplement 2.
Data Sharing Statement
