# Supplementary material for: Obstetric Characteristics and Outcomes of Gestational Carrier Pregnancies: A Systematic Review and Meta-Analysis
Source: JAMA Netw Open. 2024 Jul 23;7(7):e2422634. doi: 10.1001/jamanetworkopen.2024.22634 (PMC11267414; doi:10.1001/jamanetworkopen.2024.22634)
Supplement: Supplement 1. — eAppendix 1. Searching Keywords eAppendix 2. PICOS Criteria for Inclusion of Systematic Review eAppendix 3. The Definition of Heterogeneity eTable 1. Metadata of Comparator and Non-Comparator Studies eTable 2. Maternal Characteristics of Gestational Carrier Pregnancies (Non-Comparator Studies) eTable 3. Obstetric Outcomes Among Gestational Carrier Pregnancies (Non-Comparator Studies) eTable 4. Obstetric Outcomes in Singleton Gestational Carrier Pregnancies (Non-Comparator Studies) eTable 5. Risk of Bias Assessment for the Comparator Study eReferences. [file jamanetwopen-e2422634-s001.pdf]

Supplemental Online Content

Matsuzaki S, Masjedi AD, Matsuzaki S, et al. Obstetric characteristics and outcomes associated with gestational carrier pregnancies: a systematic review and meta-analysis. *JAMA Netw. Open*. 2024; 7(7): e2422634. doi:10.1001/jamanetworkopen.2024.22634

eAppendix 1. Searching Keywords
eAppendix 2. PICOS Criteria for Inclusion of Systematic Review
eAppendix 3. The Definition of Heterogeneity
eTable 1. Metadata of Comparator and Non-Comparator Studies
eTable 2. Maternal Characteristics of Gestational Carrier Pregnancies (Non-Comparator Studies)
eTable 3. Obstetric Outcomes Among Gestational Carrier Pregnancies (Non-Comparator Studies)
eTable 4. Obstetric Outcomes in Singleton Gestational Carrier Pregnancies (Non-Comparator Studies)
eTable 5. Risk of Bias Assessment for the Comparator Study
eReferences.

This supplemental material has been provided by the authors to give readers additional information about their work.

© 2024 Matsuzaki S et al. *JAMA Network Open*

eAppendix 1. Searching Keywords

PubMed

#1 Surrogate Mothers [MeSH]

#2 “Surrogate Mothers” [TIAB] OR “gestational surrogacy” [TIAB] OR “gestational surrogate” [TIAB] OR “gestational carrier” [TIAB] OR “Host Mother” [TIAB] OR “Gestational Mother” [TIAB]

#3 #1 OR #2

#4 Pregnancy [MeSH]

#5 Pregnancy outcome [MeSH] OR Pregnancy Complications [MeSH]

#6 “obstetrical complication” [TIAB] OR “obstetric outcome” [TIAB] OR “fetal outcome” [TIAB] OR “neonatal outcome” [TIAB] OR “perinatal outcome” [TIAB] OR “maternal outcome” [TIAB] OR “surgical outcome” [TIAB] OR “pregnancy outcome” [TIAB]

#7 Placenta previa [MeSH] OR “placenta previa" [TIAB] OR "low lying placenta" [TIAB] OR "abnormal placenta*" [TIAB]

#8 Pre-Eclampsia [MeSH] OR Preeclampsia [TIAB] OR hypertension [TIAB] OR Fetal growth retardation [MeSH] OR “fetal growth restriction” [TIAB] OR “intrauterine growth restriction” [TIAB] OR “intrauterine growth retardation” [TIAB] OR Pre-Eclampsia [MeSH] OR preeclampsia [TIAB] OR “pre-eclampsia” [TIAB]

#9 Postpartum Hemorrhage [MeSH] OR “postpartum hemorrhage” [TIAB] OR Obstetric Labor Complications [MeSH] OR “obstetric labor complications” [TIAB] OR “labor complications” [TIAB] OR Abruptio Placentae [MeSH] OR “abruptio placentae” [TIAB] OR “placental abruption” [TIAB] OR “placental abruptions” [TIAB] OR Uterine Rupture [MeSH] OR “uterine rupture” [TIAB] OR “uterine ruptures” [TIAB]

#10 Cesarean section [MeSH] OR cesarean section [TIAB] OR Cesarean delivery [TIAB] OR abdominal delivery [TIAB] OR “C section” [TIAB]

#11 #4OR #5 OR #5 OR #6 OR #7 OR #8 OR #9 OR #10

#12 #3 AND #11

Scopus

#1 “Surrogate Mothers” OR “gestational surrogacy” OR “gestational surrogate” OR “gestational carrier” OR “Host Mother” OR “Gestational Mother”

#2 (gestational OR host) W/3 (surrogate OR surrogacy OR Mother OR carrier)

#3 #1 OR #2

#4 TITLE-ABS-KEY (pregnancy OR pregnanc* OR pregnant OR gravid* OR obstet* OR postpartum* OR birth OR fetus* OR foetus* OR fetal OR foetal OR gestation OR gestations OR “obstetrical complication” OR "obstetric outcome” OR “fetal outcome” OR “neonatal outcome” OR “perinatal outcome” OR “maternal outcome” OR “surgical outcome” OR “pregnancy outcome”)

#5 TITLE-ABS-KEY (“placenta previa” OR “low lying placenta” OR “abnormal placentation”)

#6 TITLE-ABS-KEY (Preeclampsia OR “pre-eclampsia” OR hypertension OR “Fetal growth

© 2024 Matsuzaki S et al. *JAMA Network Open*

retardation” OR “fetal growth restriction” OR “intrauterine growth restriction” OR “intrauterine growth retardation” OR “placenta previa” OR "low lying placenta" OR “fetal growth restriction” OR “intrauterine growth restriction” OR “intrauterine growth retardation” OR preeclampsia OR “pre-eclampsia” OR “postpartum hemorrhage” OR “obstetric labor complications” OR “labor complications” OR “abruptio placentae” OR “placental abruption” OR “placental abruptions”)
#7 TITLE-ABS-KEY (“uterine rupture” OR “uterine ruptures” OR “dystocia” OR “dystocias” OR “breech presentation” OR “breech fetal presentation” OR “fetal malpresentation”)
#8 TITLE-ABS-KEY (“cesarean section” OR “cesarean delivery” OR “abdominal delivery” OR “C section”)
#9 #4 OR #5 OR #6 OR #7 OR #8
#10 #3 AND #9

Cochrane
#1 Surrogate Mothers [MeSH]
#2 (gestational OR host) NEAR/3 (surrogate OR surrogacy OR Mother OR carrier)
#3 #1 OR #2
#4 Pregnancy [MeSH]
#5 Pregnancy outcome [MeSH]
#6 Pregnancy Complications [MeSH]
#7 “obstetrical complication”: ab,ti,kw OR “obstetric outcome”: ab,ti,kw OR “fetal outcome”: ab,ti,kw OR “neonatal outcome”: ab,ti,kw OR “perinatal outcome”: ab,ti,kw OR “maternal outcome”: ab,ti,kw OR “surgical outcome”: ab,ti,kw OR “pregnancy outcome”: ab,ti,kw
#8 Placenta previa [MeSH]
#9 “placenta previa": ab,ti,kw OR "low lying placenta": ab,ti,kw OR "abnormal placenta*": ab,ti,kw
#10 Pre-Eclampsia [MeSH]
#11 Preeclampsia: ab,ti,kw OR hypertension: ab,ti,kw
#12 Fetal growth retardation [MeSH]
#13 “fetal growth restriction”: ab,ti,kw OR “intrauterine growth restriction”: ab,ti,kw OR “intrauterine growth retardation”: ab,ti,kw OR preeclampsia: ab,ti,kw OR “pre-eclampsia”: ab,ti,kw
#14 Postpartum Hemorrhage [MeSH]
#15 Obstetric Labor Complications [MeSH]
#16 “obstetric labor complications”: ab,ti,kw OR “labor complications”: ab,ti,kw
#17 Abruptio Placentae [MeSH]
#18 “abruptio placentae”: ab,ti,kw OR “placental abruption”: ab,ti,kw OR “placental abruptions”: ab,ti,kw
#19 Uterine Rupture [MeSH]
#20 “uterine rupture”: ab,ti,kw OR “uterine ruptures”: ab,ti,kw
#21 Dystocia [MeSH]
#22 “dystocia”: ab,ti,kw OR “dystocias”: ab,ti,kw

© 2024 Matsuzaki S et al. *JAMA Network Open*

#23 Breech Presentation [MeSH]
#24 “breech presentation”: ab,ti,kw OR “breech fetal presentation”: ab,ti,kw
#25 Cesarean section [MeSH]
#26 Cesarean section: ab,ti,kw OR Cesarean delivery: ab,ti,kw OR abdominal delivery: ab,ti,kw OR “C section”: ab,ti,kw
#27 #4 OR #5 OR #6 OR #7 OR #8 OR #9 OR #10 OR #11 OR #12 OR #13 OR #14 OR #15 OR #16 OR #17 OR #18 OR #19 OR #20 OR #21 OR #22 OR #23 OR #24 OR #25 OR #26
#28 #3 AND #27

Web Of Science
#1 ”Surrogate Mothers” OR “gestational surrogacy” OR “gestational surrogate” OR “gestational carrier” OR “Host Mother” OR “Gestational Mother”
#2 (gestational OR host) NEAR/3 (surrogate OR surrogacy OR Mother OR carrier)
#3 #1 OR #2
#4 pregnancy OR pregnanc* OR pregnant OR gravid* OR obstet* OR postpartum* OR birth OR fetus* OR foetus* OR fetal OR foetal OR gestation OR gestations OR “obstetrical complication” OR “obstetric outcome” OR “fetal outcome” OR “neonatal outcome” OR “perinatal outcome” OR “maternal outcome” OR “surgical outcome” OR “pregnancy outcome”
#5 “placenta previa” OR “low lying placenta” OR “abnormal placentation”
#6 Preeclampsia OR “pre-eclampsia” OR hypertension OR “Fetal growth retardation” OR “fetal growth restriction” OR “intrauterine growth restriction” OR “intrauterine growth retardation” OR “placenta previa” OR "low lying placenta" OR “fetal growth restriction” OR “intrauterine growth restriction” OR “intrauterine growth retardation” OR preeclampsia OR “pre-eclampsia” OR “postpartum hemorrhage” OR “obstetric labor complications” OR “labor complications” OR “abruptio placentae” OR “placental abruption” OR “placental abruptions”
#7 “uterine rupture” OR “uterine ruptures” OR “dystocia” OR “dystocias” OR “breech presentation” OR “breech fetal presentation” OR “fetal malpresentation”
#8 “cesarean section” OR “cesarean delivery” OR “abdominal delivery” OR “C section”
#9 #4 OR #5 OR #6 OR #7 OR #8
#10 #3 AND #9

© 2024 Matsuzaki S et al. *JAMA Network Open*

eAppendix 2. PICOS Criteria for Inclusion of Systematic Review

**Population**	Pregnant women
**Intervention**	Gestational carrier
**Comparison**	Gestational carrier *versus* non-gestational carrier
**Outcome**	Maternal characteristics and outcomes of gestational carrier
**Study design**	Retrospective or prospective cohort studies, case-control study, and randomized controlled trials

Abbreviation: PICOS, Patient/Population, Intervention, Comparator, Outcome, Study.

© 2024 Matsuzaki S et al. *JAMA Network Open*

eAppendix 3. The Definition of Heterogeneity

I^2 value	Heterogeneity	Analysis
0%–40%	Low	Fixed-effect
30%–60%	Moderate	Random-effect
50%–90%	Substantial	Random-effect
75%–100%	Considerable	Random-effect

According to the *Cochrane Handbook for Systematic Reviews of Interventions* (ver 6.4), heterogeneity was determined per the I^2 value.[1]

© 2024 Matsuzaki S et al. *JAMA Network Open*

eTable 1. Metadata of Comparator and Non-Comparator Studies

	Year	Location	No.	Exp (n)	Cont (n)	Exp	Cont
Comparator							
Shandley LM[2]	2023	USA	1008205	40177	968028	GC	Non-GC
Swanson K[3]	2021	USA	509376	361	509015	GC	General
				361	567	GC	Non-GC ART
Segal TR[4]	2018	USA	8384	1,009	7375	GC	Intended parent
Sunkara SK[5]	2017	IND	103160	244	87571	GC	Non-GC_Fresh
				244	15345	GC	Non-GC_Frozen
Perkins KM[6]	2016	USA	174357	3857	170500	GC	Non-GC ART
Gibbons WE[7]	2011	USA	1180	1180	n/a	GC	Non-GC ART
Non-comparator							
Attawet J[8]	2022	AUS	41	41	n/a	GC	n/a
Smith MB[9]	2021	USA	836	836	n/a	GC	n/a
Namath A[10]	2021	USA	683*	427*	156*	SET	DET
Swanson K[11]	2021	USA	361	284	77	Single	Multi
Pavlovic Z[12]	2020	USA	149	78	71	GC	Prior preg
Swanson K[13]	2020	USA	361	303	58	Meets[#]	Deviates[#]
Peters HE[14]	2018	NLD	34	34	n/a	GC	n/a
Fuchs EL[15]	2018	USA	222	204	18	GC	TS
Woo I[16]	2017	USA	397	103	294	GC	Spont
Dar S[17]	2015	CAN	133	76	57	GC_oocyte	GC_non oocyte
Dermout S[18]	2010	NLD	35	35	n/a	GC	n/a
Duffy DA[19]	2005	USA	10	10	n/a	GC	n/a
Söderström V[20]	2002	FIN	10	10	n/a	GC	n/a
Parkinson J[21]	1999	USA	95	95	n/a	GC	n/a
Corson SL[22]	1998	USA	94	94	n/a	GC	n/a
Reame NE[23]	1990	USA	37	37	n/a	GC	n/a

* Restricted to singleton pregnancies; # American Society for Reproductive Medicine guidelines. Metadata of eligible studies are shown. Abbreviations: No. total number of included cases; vs, versus; Exp (n), number of experimental group; Cont (n), number of control group, Exp, type of experimental group; Cont, type of control group; GC, gestational carrier; ART, assisted reproductive technology; General, general pregnancies; Spont, spontaneously conceived pregnancies in the same woman; Fresh, fresh embryo transfer; Frozen, frozen embryo transfer; oocyte, oocyte donation; TS, traditional surrogacy; Prior preg, prior own pregnancy; Single, singleton pregnancy; Multiple, multiple pregnancy; SET, single embryo transfer; DET, double embryo transfer; USA, United states of America; IND, India; AUS, Australia; NDL, Netherlands; FIN, Finland.

© 2024 Matsuzaki S et al. *JAMA Network Open*

eTable 2. Maternal Characteristics of Gestational Carrier Pregnancies (Non-Comparator Studies)

Author	Year	No.	Age (yr)‡	Nuli	Multi	AMA (≥ 35yr)	GDM
Attawet J[8]	2022	41	n/a	n/a	1 (2.4)	n/a	n/a
Smith MB[9]	2021	836	33.92 ± 4.61	1 (0.1)	187 (22.4)	367 (43.9)	n/a
Namath A[10]	2021	237#	n/a	n/a	19 (8.0)	n/a	n/a
Swanson K[11]	2021	361#	n/a	11 (3.0)	77 (21.3)	n/a	n/a
Pavlovic Z[12]	2020	78*	n/a	n/a	0*	n/a	1 (1.3)
Peters HE[14]	2018	34	n/a	n/a	0	n/a	n/a
Fuchs EL[15]	2018	222	32.7 ± 5.3	n/a	77 (34.7)	n/a	n/a
Woo I[16]	2017	103*,#	38.8 ± 2.1	n/a	0*	n/a	7 (6.8)
Dar S[17]	2015	133	n/a	n/a	40 (30.1)	n/a	4 (3.0)
Dermout S[18]	2010	13#	n/a	n/a	3 (23.1)	n/a	n/a
Duffy DA[19]	2005	8',#	38.7 ± 5.3	n/a	2 (28.6)	6 (75.0)	n/a
Söderström V[20]	2002	10#	36	n/a	1 (10.0)	n/a	2 (20.0)
Parkinson J[21]	1999	95§	n/a	n/a	29 (30.9)	n/a	2 (2.2)
Corson SL[22]	1998	27#	n/a	n/a	6 (16.2)	n/a	n/a
Reame NE[23]	1990	38	n/a	n/a	1 (2.6)	n/a	n/a
Cumulative data	n/a	2236	34.2	12 (1.0)	443 (21.6)	373 (44.2)	16 (3.8)

Mean (SD) or number (percentage per column) is shown. # Number of pregnancies with live births. § 4 singleton pregnancies were lost follow; ' one patient had two pregnancies; * Excluding multiple pregnancies;‡ Mean ± SD or Mean. Abbreviations: No., number of GC pregnancies; yr, years old; SD, standard deviation; GC, gestational carrier pregnancies; Nuli, nulliparous; Multi, multiple pregnancies; AMA, advanced maternal age; and GDM, gestational diabetes mellitus. In the non-comparator studies, the cumulative mean maternal age of GCs was 34.2 years (range 32.7-38.8), the cumulative rate of multifetal gestation (excluding singleton-restricted studies) reached 21.6% (443 / 2,055 patients: range 0-34.7%), and the rate of GDM was 3.8% (16 / 419 patients: range 1.3-20%). Nulliparity was detailed in two studies, with a rate of 1.0% (12 / 1,222 patients: range 0.1-3.0%).

© 2024 Matsuzaki S et al. *JAMA Network Open*

eTable 3. Obstetric Outcomes Among Gestational Carrier Pregnancies (Non-Comparator Studies)

	Year	No.	HDP	FGR	PTB	PP	IUFD	AB	LBW[&]	CD	PPH	SMM	Mortality
Attawet J[8]	2022	41	n/a	1 (2.4)	6 (14.6)	n/a	1 (2.4)	n/a	n/a	16 (39.0)	n/a	n/a	n/a
Smith MB[9]	2021	836	n/a	n/a	39 (15.1)[†]	n/a	n/a	n/a	n/a	319 (38.2)	n/a	n/a	n/a
Namath A[10]	2021	237[#]	n/a	n/a	53 (22.4)	n/a	n/a	n/a	16 (6.8)	n/a	n/a	n/a	n/a
Swanson K[11]	2021	361[#]	38 (10.5)	n/a	96 (26.6)	n/a	n/a	n/a	n/a	92 (25.5)	n/a	a	0
Pavlovic Z[12]	2020	78*	7 (9.0)	2 (2.6)	8 (10.2)	1 (1.3)	n/a	1 (1.3)	n/a	9 (11.5)	2 (2.6)	n/a	n/a
Peters HE[14]	2018	34	5 (14.7)	n/a	0	n/a	n/a	n/a	1 (2.9)	3 (8.8)	5 (14.7)	b	n/a
Fuchs EL[15]	2018	222	n/a	n/a	n/a	n/a	n/a	n/a	n/a	82 (36.9)	n/a	n/a	n/a
Woo I[16]	2017	103[*,#]	2 (1.9)	n/a	11 (10.7)	5 (4.9)	n/a	n/a	8 (7.8)	19 (19.0)	2 (1.9)	n/a	n/a
Dar S[17]	2015	133	3 (2.3)	1 (0.6)	25 (18.8)	1 (0.8)	2 (1.6)	1 (0.8)	32 (24.1)	31 (23.3)	n/a	c	n/a
Dermout S[18]	2010	13[#]	n/a	n/a	3 (23.1)	n/a	n/a	n/a	n/a	4 (30.8)	1 (7.7)	d	0
Duffy DA[19]	2005	8[',#]	n/a	n/a	2 (25.0)	n/a	n/a	n/a	2 (25.0)	3 (37.5)	n/a	e	0
Söderström V[20]	2002	10[#]	1 (10.0)	n/a	n/a	n/a	n/a	n/a	2 (20)	10 (100)	n/a	n/a	n/a
Parkinson J[21]	1999	95[§]	5 (5.5)	n/a	15 (16.5)	n/a	n/a	n/a	n/a	32 (34.0)	n/a	n/a	n/a
Corson SL[22]	1998	27[#]	n/a	n/a	3 (11.1)	n/a	n/a	n/a	n/a	n/a	n/a	n/a	n/a
Reame NE[23]	1990	38	n/a	n/a	3 (7.9)	1 (2.6)	n/a	2 (5.2)	n/a	5 (13.2)	n/a	n/a	0
Cumulative	n/a	2236	61 (7.5)	4 (1.6)	264 (18.5)	8 (2.3)	3 (1.7)	4 (1.6)	61 (11.6)	625 (31.7)	10 (5.3)	f	0

Number (percentage per column) is shown. [#] Number of pregnancies with live births. [&] in case of multiple pregnancies, one, two, or all neonates with LBW were counted as 1; [§] 4 singleton pregnancies were lost follow; ' one patient had two pregnancies; [*] Excluding multiple pregnancies; [†] Data on gestational age at delivery of GC pregnancies was available for 259 deliveries; a: Maternal death 0; ICU admission 2 (0.6%)1; Eclampsia 0; HELLP: 1 (0.3%); Transfusion 4 (1.1%); Hysterectomy 0; b: HELLP 1 (2.9%); c: Hysterectomy 1/133 (0.8%); d: Transfusion 1/13 (7.7%); e: hysterectomy 2/8 (25.0%); f: ICU admission (0.6%), eclampsia 0, HELLP syndrome 1 (0.5%), transfusion (1.3%), and hysterectomy 3 (0.6%). Abbreviations: No., total number of gestational carrier pregnancies; Cumulative, cumulative rate; SMM, severe maternal morbidity; HDP, hypertensive disorders of pregnancy; FGR, fetal growth restriction; PTB, preterm birth; PP, placenta previa; IUFD, intrauterine fetal death; AB, placenta abruption; LBW, low birth weight; CD, cesarean delivery; and PPH, postpartum hemorrhage.

The number of non-comparator studies (*n*=15) examined HDP (*n*=7), FGR (*n*=3), PTB (*n*=13), placenta previa (*n*=4), IUFD (*n*=2), placental abruption (*n*=3), LBW (*n*=5), CD (*n*=13), PPH (*n*=4), maternal mortality (*n*=4), and SMM (*n*=5). Among these, 12 studies provided specific data regarding GC singleton pregnancies. No studies have shown the rate of placenta accreta spectrum in GC pregnancies. Seven non-comparator studies examined HDP, revealing a cumulative rate of 7.5% in GCs (range 1.9-14.7%). The cumulative PTB rate across 13 non-comparator studies was 18.5% (range 0-26.6%) for the entire cohort. LBW rates were evaluated in 6 of 15 non-comparator studies, showing a cumulative rate of 11.6% (*n*=6: range 2.9-25%) in the entire cohort. Four non-comparator studies examined the rate of maternal death, with no maternal deaths observed in 420 live births to GCs.

Rates of FGR, PP, IUFD, placental abruption, and PPH were available only in non-comparator studies. The entire cohort showed the following rates: FGR, 1.6% (*n*=3; range 0.6-2.6%); PP, 2.3% (*n*=4; range 0.8-4.9%); IUFD, 1.7% (*n*=2; range 1.6-2.4%); placental abruption, 1.6% (*n*=3; range 0.8-5.2%); and PPH, 5.3% (*n*=4; range 1.9-14.7%).

© 2024 Matsuzaki S et al. *JAMA Network Open*

eTable 4. Obstetric Outcomes in Singleton Gestational Carrier Pregnancies (Non-Comparator Studies)

	Year	No.	HDP	FGR	PTB	PP	IUFD	AB	LBW#	CD	PPH
Attawet J[8]	2022	40	n/a	n/a	n/a	n/a	n/a	n/a	2 (5.0)	n/a	n/a
Smith MB[9]	2021	204†	n/a	n/a	34 (16.7)	n/a	n/a	n/a	n/a	n/a	n/a
Swanson K[11]	2021	284#	28 (9.9)	n/a	37 (13.0)	n/a	n/a	n/a	n/a	52 (18.3)	n/a
Pavlovic Z[12]	2020	78*	7 (9.0)	2 (2.6)	8 (10.2)	1 (1.3)	n/a	1 (1.3)	n/a	9 (11.5)	2 (2.6)
Peters HE[14]	2018	34	5 (14.7)	n/a	0	n/a	n/a	n/a	1 (2.9)	3 (8.8)	5 (14.7)
Woo I[16]	2017	103*,#	2 (1.9)	n/a	11 (10.7)	5 (4.9)	n/a	n/a	8 (7.8)	19 (19.0)	2 (1.9)
Dar S[17]	2015	93	2 (2.2)	0	6 (6.5)	0	1 (1.1)	1 (1.1)	11 (11.8)	n/a	n/a
Dermout S[18]	2010	10#	n/a	n/a	0	n/a	n/a	n/a	n/a	3 (30.0)	0
Duffy DA[19]	2005	6#	n/a	n/a	0	n/a	n/a	n/a	0	2 (33.3)	n/a
SöderströmV[20]	2002	9#	n/a	n/a	n/a	n/a	n/a	n/a	0	9 (100)	n/a
Parkinson J[21]	1999	65§	3 (4.9)	n/a	7 (11.5)	n/a	n/a	n/a	n/a	13 (21.3)	n/a
Reame NE[23]	1990	37	n/a	n/a	2 (5.4)	1 (2.7)	n/a	2 (5.4)	n/a	5 (13.5)	n/a
Cumulative	n/a	963	47 (7.2)	2 (1.2)	105 (11.5)	7 (2.3)	1 (1.1)	4 (1.9)	22 (7.7)	115 (18.4)	9 (4.4)

Number (percentage per column) is shown. † Estimated by the authors. # in case of multiple pregnancies, one, two, or all neonates with LBW were counted as 1; § 4 singleton pregnancies were lost follow; * Excluding multiple pregnancies. Abbreviations: No., total number of singleton gestational carrier pregnancies; HDP, hypertensive disorders of pregnancy; FGR, fetal growth restriction; PTB, preterm birth; PP, placenta previa; IUFD, intrauterine fetal death; AB, placenta abruption; LBW, low birth weight; CD, cesarean delivery; and PPH, postpartum hemorrhage. Six non-comparator studies examined HDP in singleton GC pregnancies, revealing a cumulative rate was in 7.2% (range 1.9-14.7%). The cumulative PTB rate across 10 non-comparator studies was 11.5% (range 0-16.7%) in singleton pregnancies. LBW rates in GC singleton pregnancies were evaluated in 6 of 15 non-comparator studies, 7.7% (range 0-11.8%) in singleton pregnancies. Rates of FGR, PP, IUFD, placental abruption, and PPH were available only in non-comparator studies. In singleton pregnancies, the rates were as follows: FGR, 1.2% (*n*=2; range 0-2.6%); placenta previa, 2.3% (*n*=4; range 0-4.9%); IUFD, 1.1% (*n*=1); placental abruption, 1.9% (*n*=3; range 1.1-5,4%); and PPH, 4.4% (*n*=4; range 1.9-14.7%).

eTable 5. Risk of Bias Assessment for the Comparator Study

Authors	Confounding	Selection	Classification of intervention	Deviations from interventions	Missing data	Measurement of outcomes	Reported results	Overall bias
Shandley LM [2]	● (purple)	● (purple)	● (purple)	● (purple)	● (purple)	● (purple)	● (purple)	● (purple)
Swanson K [3]	● (purple)	● (purple)	● (purple)	● (purple)	● (black)	● (purple)	● (purple)	● (purple)
Segal TR [4]	● (purple)	● (red)	● (red)	● (purple)	● (black)	● (purple)	● (purple)	● (red)
Sunkara SK [5]	● (purple)	● (purple)	● (purple)	● (purple)	● (yellow)	● (purple)	● (yellow)	● (purple)
Perkins KM [6]	● (purple)	● (purple)	● (purple)	● (purple)	● (yellow)	● (purple)	● (purple)	● (purple)
Gibbons WE [7]	● (red)	● (red)	● (red)	● (purple)	● (black)	● (red)	● (purple)	● (red)

Risk of bias assessment was performed using the Risk Of Bias In Non-randomized Studies–of Interventions tool (ROBINS-I).[24-26]

● (orange) Low risk of bias (the study is comparable to a well-performed randomized trial with regard to this domain)
● (purple) Moderate risk of bias (the study is sound for a non-randomized study with regard to this domain but cannot be considered comparable to a well-performed randomized trial)
● (dark red) Serious risk of bias (the study has some important problems in this domain)
● (bright red) Critical risk of bias (the study is too problematic in this domain to provide any useful evidence on the effects of intervention.
● (black) No information on how to base a judgment on the risk of bias for this domain.

© 2024 Matsuzaki S et al. *JAMA Network Open*

eReferences

1. Cochrane Higgins JPT, Thomas J, Chandler J, Cumpston M, Li T, Page MJ, Welch VA (editors). Cochrane Handbook for Systematic Reviews of Interventions version 6.4 (updated August 2023). Cochrane, 2023. Available from www.training.cochrane.org/handbook. (accessed 02/20/2024).
2. Shandley LM, DeSantis CE, Lee JC, Kawwass JF, Hipp HS. Trends and Outcomes of Assisted Reproductive Technology Cycles Using a Gestational Carrier Between 2014 and 2020. *Jama*. Nov 7 2023;330(17):1691-1694. doi:10.1001/jama.2023.11023
3. Swanson K, Letourneau JM, Kuppermann M, Einerson BD. Obstetric morbidity in gestational carrier pregnancies: a population-based study. *Journal of assisted reproduction and genetics*. Jan 2021;38(1):177-183. doi:10.1007/s10815-020-02000-4
4. Segal TR, Kim K, Mumford SL, Goldfarb JM, Weinerman RS. How much does the uterus matter? Perinatal outcomes are improved when donor oocyte embryos are transferred to gestational carriers compared to intended parent recipients. *Fertility and sterility*. Oct 2018;110(5):888-895. doi:10.1016/j.fertnstert.2018.06.015
5. Sunkara SK, Antonisamy B, Selliah HY, Kamath MS. Perinatal outcomes after gestational surrogacy versus autologous IVF: analysis of national data. *Reproductive biomedicine online*. Dec 2017;35(6):708-714. doi:10.1016/j.rbmo.2017.08.024
6. Perkins KM, Boulet SL, Jamieson DJ, Kissin DM. Trends and outcomes of gestational surrogacy in the United States. *Fertility and sterility*. Aug 2016;106(2):435-442.e2. doi:10.1016/j.fertnstert.2016.03.050
7. Gibbons WE, Cedars M, Ness RB. Toward understanding obstetrical outcome in advanced assisted reproduction: varying sperm, oocyte, and uterine source and diagnosis. *Fertility and sterility*. Apr 2011;95(5):1645-9.e1. doi:10.1016/j.fertnstert.2010.11.029
8. Attawet J, Wang A, Li Z, Johnson L, Hammarberg K, Sullivan E. Cumulative live birth rates among gestational surrogates in altruistic surrogacy arrangements. *Human fertility (Cambridge, England)*. Apr 2022;25(2):329-336. doi:10.1080/14647273.2020.1794062
9. Smith MB, Mandelbaum RS, McGinnis LK, Paulson RJ. Examining pre-term birth and cesarean section rates in gestational carrier pregnancies. *Journal of assisted reproduction and genetics*. Oct 2021;38(10):2707-2712. doi:10.1007/s10815-021-02296-w
10. Namath A, Jahandideh S, Devine K, O'Brien JE, Stillman RJ. Gestational carrier pregnancy outcomes from frozen embryo transfer depending on the number of embryos transferred and preimplantation genetic testing: a retrospective analysis. *Fertility and sterility*. Jun 2021;115(6):1471-1477. doi:10.1016/j.fertnstert.2021.01.010
11. Swanson K, Debbink M, Letourneau JM, Kuppermann M, Einerson BD. Association of multifetal gestation with obstetric and neonatal outcomes in gestational carrier pregnancies. *Journal of assisted reproduction and genetics*. Mar 2021;38(3):661-667. doi:10.1007/s10815-020-02034-8
12. Pavlovic Z, Hammer KC, Raff M, et al. Comparison of perinatal outcomes between spontaneous vs. commissioned cycles in gestational carriers for single and same-sex male intended parents. *Journal of assisted reproduction and genetics*. Apr 2020;37(4):953-962. doi:10.1007/s10815-020-01728-3
13. Swanson K, Letourneau JM, Kuppermann M, Einerson BD. Association of Obstetric and Neonatal Outcomes With Deviation From Guidelines for Gestational Carriers. *Obstetrics and gynecology*. Aug 2020;136(2):387-393. doi:10.1097/aog.0000000000003918
14. Peters HE, Schats R, Verhoeven MO, et al. Gestational surrogacy: results of 10 years of experience in the Netherlands. *Reproductive biomedicine online*. Dec 2018;37(6):725-731. doi:10.1016/j.rbmo.2018.09.017
15. Fuchs EL, Berenson AB. Outcomes for Gestational Carriers Versus Traditional Surrogates in the United States. *Journal of women's health (2002)*. May 2018;27(5):640-645. doi:10.1089/jwh.2017.6540
16. Woo I, Hindoyan R, Landay M, et al. Perinatal outcomes after natural conception versus in vitro fertilization (IVF) in gestational surrogates: a model to evaluate IVF treatment versus maternal effects. *Fertility and sterility*. Dec 2017;108(6):993-998. doi:10.1016/j.fertnstert.2017.09.014
17. Dar S, Lazer T, Swanson S, et al. Assisted reproduction involving gestational surrogacy: an analysis of the medical, psychosocial and legal issues: experience from a large surrogacy program. *Human reproduction (Oxford, England)*. Feb 2015;30(2):345-52. doi:10.1093/humrep/deu333
18. Dermout S, van de Wiel H, Heintz P, Jansen K, Ankum W. Non-commercial surrogacy: an account of patient management in the first Dutch Centre for IVF Surrogacy, from 1997 to 2004. *Human reproduction (Oxford, England)*. Feb 2010;25(2):443-9. doi:10.1093/humrep/dep410
19. Duffy DA, Nulsen JC, Maier DB, Engmann L, Schmidt D, Benadiva CA. Obstetrical complications in gestational carrier pregnancies. *Fertility and sterility*. Mar 2005;83(3):749-54.

© 2024 Matsuzaki S et al. *JAMA Network Open*

doi:10.1016/j.fertnstert.2004.08.023
20. Söderström-Anttila V, Blomqvist T, Foudila T, et al. Experience of in vitro fertilization surrogacy in Finland. *Acta obstetricia et gynecologica Scandinavica*. Aug 2002;81(8):747-52.
21. Parkinson J, Tran C, Tan T, Nelson J, Batzofin J, Serafini P. Perinatal outcome after in-vitro fertilization-surrogacy. *Human reproduction (Oxford, England)*. Mar 1999;14(3):671-6. doi:10.1093/humrep/14.3.671
22. Corson SL, Kelly M, Braverman AM, English ME. Gestational carrier pregnancy. *Fertility and sterility*. Apr 1998;69(4):670-4. doi:10.1016/s0015-0282(98)00020-x
23. Reame NE, Parker PJ. Surrogate pregnancy: clinical features of forty-four cases. *American journal of obstetrics and gynecology*. May 1990;162(5):1220-5. doi:10.1016/0002-9378(90)90022-y
24. Sterne JA, Hernan MA, Reeves BC, et al. ROBINS-I: a tool for assessing risk of bias in non-randomised studies of interventions. *BMJ*. Oct 12 2016;355:i4919. doi:10.1136/bmj.i4919
25. Danna SM, Graham E, Burns RJ, Deschenes SS, Schmitz N. Association between Depressive Symptoms and Cognitive Function in Persons with Diabetes Mellitus: A Systematic Review. *PLoS One*. 2016;11(8):e0160809. doi:10.1371/journal.pone.0160809
26. ROBINS-I detailed guidance (2016). https://www.riskofbias.info/welcome/home/current-version-of-robins-i/robins-i-detailed-guidance-2016. (accessed 02/20/2024).

© 2024 Matsuzaki S et al. *JAMA Network Open*
