# Supplementary material for: Obstetric Characteristics and Outcomes of Gestational Carrier Pregnancies: A Systematic Review and Meta-Analysis
Source: JAMA Netw Open. 2024 Jul 23;7(7):e2422634. doi: 10.1001/jamanetworkopen.2024.22634 (PMC11267414; doi:10.1001/jamanetworkopen.2024.22634)
Supplement: Supplement 2. — Data Sharing Statement [file jamanetwopen-e2422634-s002.pdf]

Data Sharing Statement

Matsuzaki. Obstetric Characteristics and Outcomes of Gestational Carrier Pregnancies. *JAMA Netw Open*. Published July 23, 2024. doi:10.1001/jamanetworkopen.2024.22634

Data
Data available: No

Additional Information
Explanation for why data not available: The data on which this study is based on the published articles.
